# Bis(μ_2_-quinoline-2-carboxyl­ato)-κ^3^ *N*,*O* ^1^:*O* ^1^;κ^3^ *O* ^1^:*N*,*O* ^1^-bis­[(acetato-κ*O*)(ethanol-κ*O*)lead(II)]

**DOI:** 10.1107/S1600536811002509

**Published:** 2011-01-29

**Authors:** Ezzatollah Najafi, Mostafa M. Amini, Seik Weng Ng

**Affiliations:** a Department of Chemistry, General Campus, Shahid Beheshti University, Tehran 1983963113, Iran; b Department of Chemistry, University of Malaya, 50603 Kuala Lumpur, Malaysia

## Abstract

In the centrosymmetric dinuclear title compound, [Pb_2_(C_10_H_6_NO_2_)_2_(CH_3_COO)_2_(C_2_H_5_OH)_2_], one O atom of the carboxyl­ate group of the quinoline-2-carboxyl­ate anion connects the two Pb^II^ atoms. The Pb^II^ atom is surrounded by four O atoms and one N atom in a Ψ-octa­hedral PbO_4_N*E* geometry (*E* is the electron lone pair). Two longer Pb⋯O inter­actions distort the geometry towards a Ψ-square-anti­prism. Inter­molecular O—H⋯O hydrogen bonds link the mol­ecules.

## Related literature

For the analogous methanol-coordinated compound, see: Mohammadnezhad *et al.* (2010 ▶).
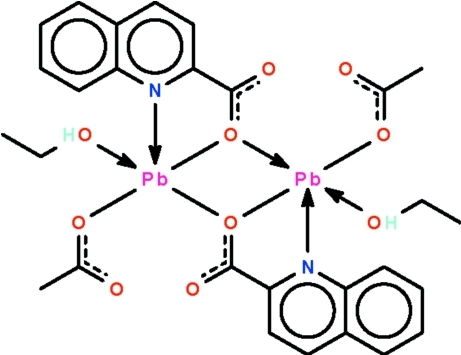


## Experimental

### 

#### Crystal data

[Pb_2_(C_10_H_6_NO_2_)_2_(C_2_H_3_O_2_)_2_(C_2_H_6_O)_2_]
*M* *_r_* = 968.92
Monoclinic, 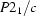

*a* = 7.3419 (1) Å
*b* = 8.4004 (1) Å
*c* = 23.8008 (4) Å
β = 93.722 (1)°
*V* = 1464.82 (4) Å^3^
*Z* = 2
Mo *K*α radiation
μ = 11.54 mm^−1^
*T* = 100 K
0.20 × 0.20 × 0.05 mm

#### Data collection

Agilent Technologies SuperNova Dual diffractometer with an Atlas detector
Absorption correction: multi-scan (*CrysAlis PRO*; Agilent Technologies, 2010 ▶) *T*_min_ = 0.206, *T*_max_ = 0.596
12668 measured reflections
3314 independent reflections
3010 reflections with *I* > 2σ(*I*)
*R*_int_ = 0.049

#### Refinement

*R*[*F* ^2^ > 2σ(*F* ^2^)] = 0.029
*wR*(*F* ^2^) = 0.073
*S* = 1.08
3314 reflections
192 parameters
H-atom parameters constrained
Δρ_max_ = 1.21 e Å^−3^
Δρ_min_ = −1.57 e Å^−3^

### 

Data collection: *CrysAlis PRO* (Agilent Technologies, 2010 ▶); cell refinement: *CrysAlis PRO*; data reduction: *CrysAlis PRO*; program(s) used to solve structure: *SHELXS97* (Sheldrick, 2008 ▶); program(s) used to refine structure: *SHELXL97* (Sheldrick, 2008 ▶); molecular graphics: *X-SEED* (Barbour, 2001 ▶); software used to prepare material for publication: *publCIF* (Westrip, 2010 ▶).

## Supplementary Material

Crystal structure: contains datablocks global, I. DOI: 10.1107/S1600536811002509/bt5469sup1.cif

Structure factors: contains datablocks I. DOI: 10.1107/S1600536811002509/bt5469Isup2.hkl

Additional supplementary materials:  crystallographic information; 3D view; checkCIF report

## Figures and Tables

**Table 1:** Selected bond lengths (Å)

Pb1—O1	2.377 (4)
Pb1—O3	2.384 (4)
Pb1—O1^i^	2.500 (3)
Pb1—N1	2.645 (4)
Pb1—O5	2.694 (4)
Pb1—O4	2.763 (3)
Pb1—O2^ii^	3.096 (4)

Symmetry codes: (i) 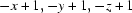; (ii) 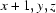.

**Table 2:** Hydrogen-bond geometry (Å, °)

*D*—H⋯*A*	*D*—H	H⋯*A*	*D*⋯*A*	*D*—H⋯*A*
O5—H5⋯O3^i^	0.84	2.36	2.710 (5)	106

Symmetry code: (i) 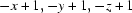.

**Fig. 1.**
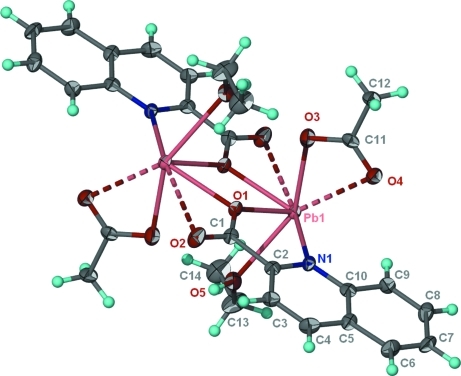
Anisotropic displacement ellipsoid plot (Barbour, 2001) of the title compound at the 70% probability level; hydrogen atoms are drawn as spheres of arbitrary radius. The longer interactions that raise the coordination number are shown as dashed lines.

## supplementary crystallographic information

### Comment

A previous study of the methanol adduct of the title dinuclear compound, [Pb(C_10_H_6_NO_2_)(C_2_H_3_O_2_)(CH_3_OH)_2_]_2_, has found a *Ψ*-octahedral geometry for the lead(II) atom. Two longer Pb···O interactions distort the geometry towards a *Ψ*-square-antiprism (Mohammadnezhad *et al.*, 2010). Replacing the methanol solvent system by ethanol leads to the analogous ethanol adduct (Scheme I, Fig. 1), which has a similar structure.

### Experimental

Lead(II) acetate (1 mmol) and quinoline-2-carboxylic acid (1 mmol) were loaded into a convection tube; the tube was filled with dry ethanol and kept at 333 K. Colorless crystals were collected from the side arm after several days.

### Refinement

H atoms were placed in calculated positions [C—H 0.95 to 0.98, O—H 0.84 Å, *U*_iso_(H) 1.2 to 1.5*U*_eq_(C, O)] and were included in the refinement in the riding model approximation.

The final difference Fourier map had a peak/hole in the vicinity of Pb1.

### Crystal data

**Table:**

[Pb_2_(C_10_H_6_NO_2_)_2_(C_2_H_3_O_2_)_2_(C_2_H_6_O)_2_]	*F*(000) = 912
*M**_r_* = 968.92	*D*_x_ = 2.197 Mg m^−^^3^
Monoclinic, *P*2_1_/*c*	Mo *K*α radiation, λ = 0.71073 Å
Hall symbol: -P 2ybc	Cell parameters from 7627 reflections
*a* = 7.3419 (1) Å	θ = 2.4–29.3°
*b* = 8.4004 (1) Å	µ = 11.54 mm^−^^1^
*c* = 23.8008 (4) Å	*T* = 100 K
β = 93.722 (1)°	Prim, colorless
*V* = 1464.82 (4) Å^3^	0.20 × 0.20 × 0.05 mm
*Z* = 2	

### Data collection

**Table:**

Agilent Technologies SuperNova Dual diffractometer with an Atlas detector	3314 independent reflections
Radiation source: SuperNova (Mo) X-ray Source	3010 reflections with *I* > 2σ(*I*)
Mirror	*R*_int_ = 0.049
Detector resolution: 10.4041 pixels mm^-1^	θ_max_ = 27.5°, θ_min_ = 2.6°
ω scan	*h* = −9→9
Absorption correction: multi-scan (*CrysAlis PRO*; Agilent Technologies, 2010)	*k* = −10→10
*T*_min_ = 0.206, *T*_max_ = 0.596	*l* = −30→30
12668 measured reflections	

### Refinement

**Table:**

Refinement on *F*^2^	Primary atom site location: structure-invariant direct methods
Least-squares matrix: full	Secondary atom site location: difference Fourier map
*R*[*F*^2^ > 2σ(*F*^2^)] = 0.029	Hydrogen site location: inferred from neighbouring sites
*wR*(*F*^2^) = 0.073	H-atom parameters constrained
*S* = 1.08	*w* = 1/[σ^2^(*F*_o_^2^) + (0.035*P*)^2^ + 1.3471*P*] where *P* = (*F*_o_^2^ + 2*F*_c_^2^)/3
3314 reflections	(Δ/σ)_max_ = 0.001
192 parameters	Δρ_max_ = 1.21 e Å^−^^3^
0 restraints	Δρ_min_ = −1.57 e Å^−^^3^

### Fractional atomic coordinates and isotropic or equivalent isotropic displacement parameters (Å^2^)

**Table:**

	*x*	*y*	*z*	*U*_iso_*/*U*_eq_	
Pb1	0.72555 (2)	0.46532 (2)	0.554759 (7)	0.01208 (8)	
O1	0.4023 (5)	0.4525 (4)	0.54078 (14)	0.0149 (7)	
O2	0.1288 (5)	0.3557 (5)	0.55875 (15)	0.0225 (8)	
O3	0.6357 (5)	0.7354 (5)	0.56638 (14)	0.0225 (8)	
O4	0.7982 (5)	0.6708 (4)	0.64405 (14)	0.0215 (8)	
O5	0.6307 (5)	0.1781 (4)	0.51184 (13)	0.0206 (8)	
H5	0.5230	0.1444	0.5119	0.031*	
N1	0.5510 (5)	0.3454 (5)	0.63884 (16)	0.0134 (8)	
C1	0.2921 (7)	0.3768 (6)	0.57117 (19)	0.0144 (10)	
C2	0.3771 (7)	0.3126 (6)	0.62685 (19)	0.0145 (10)	
C3	0.2694 (7)	0.2289 (6)	0.66278 (19)	0.0168 (10)	
H3	0.1453	0.2066	0.6518	0.020*	
C4	0.3433 (7)	0.1795 (7)	0.7138 (2)	0.0199 (11)	
H4	0.2712	0.1234	0.7390	0.024*	
C5	0.5280 (7)	0.2127 (6)	0.72855 (19)	0.0162 (10)	
C6	0.6171 (7)	0.1640 (7)	0.7808 (2)	0.0211 (11)	
H6	0.5501	0.1091	0.8076	0.025*	
C7	0.7974 (7)	0.1958 (6)	0.7925 (2)	0.0206 (11)	
H7	0.8559	0.1611	0.8271	0.025*	
C8	0.8983 (7)	0.2801 (6)	0.7536 (2)	0.0208 (11)	
H8	1.0239	0.3017	0.7624	0.025*	
C9	0.8177 (7)	0.3308 (6)	0.70336 (19)	0.0168 (10)	
H9	0.8862	0.3887	0.6777	0.020*	
C10	0.6310 (7)	0.2963 (6)	0.68997 (19)	0.0155 (10)	
C11	0.7125 (7)	0.7694 (6)	0.61446 (19)	0.0155 (10)	
C12	0.6856 (8)	0.9361 (6)	0.6361 (2)	0.0225 (12)	
H12A	0.8021	0.9774	0.6524	0.034*	
H12B	0.5964	0.9343	0.6650	0.034*	
H12C	0.6406	1.0049	0.6050	0.034*	
C13	0.7704 (9)	0.0864 (8)	0.4891 (2)	0.0311 (14)	
H13A	0.7238	−0.0224	0.4810	0.037*	
H13B	0.8746	0.0775	0.5175	0.037*	
C14	0.8359 (8)	0.1573 (8)	0.4361 (2)	0.0315 (14)	
H14A	0.9244	0.0853	0.4204	0.047*	
H14B	0.8941	0.2602	0.4448	0.047*	
H14C	0.7319	0.1727	0.4087	0.047*	

### Atomic displacement parameters (Å^2^)

**Table:**

	*U*^11^	*U*^22^	*U*^33^	*U*^12^	*U*^13^	*U*^23^
Pb1	0.00993 (11)	0.01418 (12)	0.01215 (12)	0.00129 (7)	0.00073 (7)	0.00054 (6)
O1	0.0135 (18)	0.0172 (19)	0.0138 (17)	0.0006 (14)	−0.0009 (14)	0.0015 (13)
O2	0.0117 (18)	0.032 (2)	0.0239 (18)	0.0019 (16)	−0.0002 (14)	0.0063 (16)
O3	0.024 (2)	0.022 (2)	0.0209 (18)	0.0037 (17)	−0.0060 (15)	−0.0039 (16)
O4	0.028 (2)	0.0179 (19)	0.0179 (17)	0.0039 (17)	−0.0045 (15)	−0.0008 (15)
O5	0.0188 (19)	0.0208 (19)	0.0218 (18)	0.0037 (16)	−0.0012 (14)	−0.0045 (15)
N1	0.013 (2)	0.014 (2)	0.0129 (19)	0.0040 (17)	−0.0012 (15)	−0.0020 (16)
C1	0.012 (2)	0.016 (3)	0.015 (2)	0.004 (2)	0.0023 (18)	−0.0019 (19)
C2	0.016 (2)	0.013 (2)	0.015 (2)	0.004 (2)	0.0026 (18)	−0.0019 (19)
C3	0.015 (2)	0.016 (3)	0.020 (2)	−0.001 (2)	0.0040 (19)	0.002 (2)
C4	0.020 (3)	0.020 (3)	0.021 (2)	0.000 (2)	0.008 (2)	0.005 (2)
C5	0.018 (3)	0.017 (3)	0.014 (2)	0.006 (2)	0.0017 (19)	−0.0030 (19)
C6	0.028 (3)	0.024 (3)	0.012 (2)	0.002 (2)	0.005 (2)	0.003 (2)
C7	0.026 (3)	0.021 (3)	0.014 (2)	0.006 (2)	−0.002 (2)	0.004 (2)
C8	0.018 (3)	0.025 (3)	0.019 (2)	0.007 (2)	−0.003 (2)	−0.004 (2)
C9	0.018 (3)	0.018 (3)	0.015 (2)	−0.002 (2)	0.0029 (19)	0.000 (2)
C10	0.019 (3)	0.011 (2)	0.017 (2)	0.005 (2)	0.0023 (19)	−0.0042 (19)
C11	0.014 (2)	0.013 (2)	0.020 (2)	−0.004 (2)	0.0050 (19)	0.000 (2)
C12	0.032 (3)	0.012 (3)	0.023 (3)	0.003 (2)	−0.002 (2)	−0.003 (2)
C13	0.030 (3)	0.031 (3)	0.034 (3)	0.019 (3)	0.007 (3)	0.003 (3)
C14	0.024 (3)	0.039 (4)	0.032 (3)	0.009 (3)	0.008 (2)	0.003 (3)

### Geometric parameters (Å, °)

**Table:**

Pb1—O1	2.377 (4)	C4—H4	0.9500
Pb1—O3	2.384 (4)	C5—C10	1.414 (7)
Pb1—O1^i^	2.500 (3)	C5—C6	1.427 (7)
Pb1—N1	2.645 (4)	C6—C7	1.362 (8)
Pb1—O5	2.694 (4)	C6—H6	0.9500
Pb1—O4	2.763 (3)	C7—C8	1.413 (7)
Pb1—O2^ii^	3.096 (4)	C7—H7	0.9500
O1—C1	1.288 (6)	C8—C9	1.367 (7)
O1—Pb1^i^	2.500 (3)	C8—H8	0.9500
O2—C1	1.229 (6)	C9—C10	1.417 (7)
O3—C11	1.275 (6)	C9—H9	0.9500
O4—C11	1.233 (6)	C11—C12	1.510 (7)
O5—C13	1.418 (6)	C12—H12A	0.9800
O5—H5	0.8400	C12—H12B	0.9800
N1—C2	1.319 (6)	C12—H12C	0.9800
N1—C10	1.380 (6)	C13—C14	1.502 (8)
C1—C2	1.526 (7)	C13—H13A	0.9900
C2—C3	1.392 (7)	C13—H13B	0.9900
C3—C4	1.362 (7)	C14—H14A	0.9800
C3—H3	0.9500	C14—H14B	0.9800
C4—C5	1.407 (7)	C14—H14C	0.9800
			
O1—Pb1—O3	77.18 (12)	C3—C4—H4	120.5
O1—Pb1—O1^i^	64.70 (14)	C5—C4—H4	120.5
O3—Pb1—O1^i^	75.67 (11)	C4—C5—C10	119.0 (4)
O1—Pb1—N1	63.92 (12)	C4—C5—C6	122.7 (5)
O3—Pb1—N1	97.09 (12)	C10—C5—C6	118.3 (5)
O1^i^—Pb1—N1	128.43 (12)	C7—C6—C5	120.4 (5)
O1—Pb1—O5	71.05 (11)	C7—C6—H6	119.8
O3—Pb1—O5	146.07 (12)	C5—C6—H6	119.8
O1^i^—Pb1—O5	80.20 (11)	C6—C7—C8	120.5 (5)
N1—Pb1—O5	79.62 (11)	C6—C7—H7	119.7
O1—Pb1—O4	106.08 (11)	C8—C7—H7	119.7
O3—Pb1—O4	50.00 (11)	C9—C8—C7	120.9 (5)
O1^i^—Pb1—O4	124.72 (10)	C9—C8—H8	119.5
N1—Pb1—O4	74.61 (11)	C7—C8—H8	119.5
O5—Pb1—O4	152.06 (10)	C8—C9—C10	119.4 (5)
O1—Pb1—O2^ii^	159.18 (11)	C8—C9—H9	120.3
O3—Pb1—O2^ii^	123.42 (12)	C10—C9—H9	120.3
O1^i^—Pb1—O2^ii^	114.32 (10)	N1—C10—C5	120.4 (4)
N1—Pb1—O2^ii^	111.89 (10)	N1—C10—C9	119.2 (4)
O5—Pb1—O2^ii^	88.18 (11)	C5—C10—C9	120.4 (4)
O4—Pb1—O2^ii^	91.48 (10)	O4—C11—O3	122.8 (5)
C1—O1—Pb1	127.0 (3)	O4—C11—C12	120.1 (4)
C1—O1—Pb1^i^	115.8 (3)	O3—C11—C12	117.0 (4)
Pb1—O1—Pb1^i^	115.30 (14)	C11—C12—H12A	109.5
C11—O3—Pb1	102.0 (3)	C11—C12—H12B	109.5
C11—O4—Pb1	85.1 (3)	H12A—C12—H12B	109.5
C13—O5—Pb1	117.1 (4)	C11—C12—H12C	109.5
C13—O5—H5	121.4	H12A—C12—H12C	109.5
Pb1—O5—H5	121.4	H12B—C12—H12C	109.5
C2—N1—C10	118.6 (4)	O5—C13—C14	112.5 (5)
C2—N1—Pb1	115.2 (3)	O5—C13—H13A	109.1
C10—N1—Pb1	125.6 (3)	C14—C13—H13A	109.1
O2—C1—O1	125.1 (4)	O5—C13—H13B	109.1
O2—C1—C2	119.7 (4)	C14—C13—H13B	109.1
O1—C1—C2	115.2 (4)	H13A—C13—H13B	107.8
N1—C2—C3	123.6 (4)	C13—C14—H14A	109.5
N1—C2—C1	116.7 (4)	C13—C14—H14B	109.5
C3—C2—C1	119.7 (4)	H14A—C14—H14B	109.5
C4—C3—C2	119.5 (5)	C13—C14—H14C	109.5
C4—C3—H3	120.3	H14A—C14—H14C	109.5
C2—C3—H3	120.3	H14B—C14—H14C	109.5
C3—C4—C5	118.9 (5)		
			
O3—Pb1—O1—C1	−116.7 (4)	O4—Pb1—N1—C10	−60.4 (4)
O1^i^—Pb1—O1—C1	163.3 (4)	O2^ii^—Pb1—N1—C10	24.9 (4)
N1—Pb1—O1—C1	−12.1 (3)	Pb1—O1—C1—O2	−170.1 (4)
O5—Pb1—O1—C1	75.4 (4)	Pb1^i^—O1—C1—O2	−6.8 (6)
O4—Pb1—O1—C1	−75.4 (4)	Pb1—O1—C1—C2	10.8 (6)
O2^ii^—Pb1—O1—C1	71.1 (5)	Pb1^i^—O1—C1—C2	174.0 (3)
O3—Pb1—O1—Pb1^i^	80.02 (15)	C10—N1—C2—C3	−0.8 (7)
O1^i^—Pb1—O1—Pb1^i^	0.0	Pb1—N1—C2—C3	170.7 (4)
N1—Pb1—O1—Pb1^i^	−175.37 (19)	C10—N1—C2—C1	176.9 (4)
O5—Pb1—O1—Pb1^i^	−87.89 (15)	Pb1—N1—C2—C1	−11.6 (5)
O4—Pb1—O1—Pb1^i^	121.29 (13)	O2—C1—C2—N1	−177.0 (4)
O2^ii^—Pb1—O1—Pb1^i^	−92.2 (3)	O1—C1—C2—N1	2.2 (6)
O1—Pb1—O3—C11	126.0 (3)	O2—C1—C2—C3	0.8 (7)
O1^i^—Pb1—O3—C11	−167.2 (3)	O1—C1—C2—C3	180.0 (4)
N1—Pb1—O3—C11	64.9 (3)	N1—C2—C3—C4	1.2 (8)
O5—Pb1—O3—C11	146.8 (3)	C1—C2—C3—C4	−176.4 (5)
O4—Pb1—O3—C11	1.8 (3)	C2—C3—C4—C5	−0.8 (7)
O2^ii^—Pb1—O3—C11	−57.3 (3)	C3—C4—C5—C10	0.0 (7)
O1—Pb1—O4—C11	−59.0 (3)	C3—C4—C5—C6	−179.4 (5)
O3—Pb1—O4—C11	−1.9 (3)	C4—C5—C6—C7	178.4 (5)
O1^i^—Pb1—O4—C11	11.1 (3)	C10—C5—C6—C7	−0.9 (8)
N1—Pb1—O4—C11	−115.3 (3)	C5—C6—C7—C8	1.2 (8)
O5—Pb1—O4—C11	−138.7 (3)	C6—C7—C8—C9	−0.3 (8)
O2^ii^—Pb1—O4—C11	132.4 (3)	C7—C8—C9—C10	−0.9 (8)
O1—Pb1—O5—C13	164.3 (3)	C2—N1—C10—C5	−0.1 (7)
O3—Pb1—O5—C13	142.8 (3)	Pb1—N1—C10—C5	−170.6 (3)
O1^i^—Pb1—O5—C13	97.8 (3)	C2—N1—C10—C9	180.0 (4)
N1—Pb1—O5—C13	−129.9 (3)	Pb1—N1—C10—C9	9.4 (6)
O4—Pb1—O5—C13	−107.0 (4)	C4—C5—C10—N1	0.4 (7)
O2^ii^—Pb1—O5—C13	−17.3 (3)	C6—C5—C10—N1	179.8 (4)
O1—Pb1—N1—C2	11.7 (3)	C4—C5—C10—C9	−179.6 (5)
O3—Pb1—N1—C2	83.6 (3)	C6—C5—C10—C9	−0.2 (7)
O1^i^—Pb1—N1—C2	6.3 (4)	C8—C9—C10—N1	−178.9 (5)
O5—Pb1—N1—C2	−62.2 (3)	C8—C9—C10—C5	1.1 (7)
O4—Pb1—N1—C2	128.7 (3)	Pb1—O4—C11—O3	3.2 (5)
O2^ii^—Pb1—N1—C2	−146.0 (3)	Pb1—O4—C11—C12	179.6 (4)
O1—Pb1—N1—C10	−177.5 (4)	Pb1—O3—C11—O4	−3.8 (5)
O3—Pb1—N1—C10	−105.5 (4)	Pb1—O3—C11—C12	179.7 (4)
O1^i^—Pb1—N1—C10	177.2 (3)	Pb1—O5—C13—C14	−68.5 (6)
O5—Pb1—N1—C10	108.7 (4)		

Symmetry codes: (i) −*x*+1, −*y*+1, −*z*+1; (ii) *x*+1, *y*, *z*.

### Hydrogen-bond geometry (Å, °)

**Table:**

*D*—H···*A*	*D*—H	H···*A*	*D*···*A*	*D*—H···*A*
O5—H5···O3^i^	0.84	2.36	2.710 (5)	106

Symmetry codes: (i) −*x*+1, −*y*+1, −*z*+1.
